# Persistent intercritical microvascular abnormalities in primary erythromelalgia: a quantitative nailfold capillaroscopy observation^؆^

**DOI:** 10.1016/j.abd.2026.501480

**Published:** 2026-10-30

**Authors:** Angelo Nigro

**Affiliations:** Department of Rheumatology of Lucania, UOSD of Rheumatology, “Madonna delle Grazie” Hospital, ASM Basilicata, Matera, Italy

Dear Editor,

Primary erythromelalgia is still poorly understood. Diagnosis rests on clinical criteria, and the disorder is generally regarded as a functional disturbance of cutaneous microcirculation. Nailfold Videocapillaroscopy (NVC) has no established role in current diagnostic frameworks, published case-level data are heterogeneous, and most reports of intercritical NVC have been unremarkable.^1^ The classic experimental work by Mørk and colleagues, now more than twenty years old, documented reduced capillary density during heat-induced attacks and proposed arteriovenous shunting as the underlying mechanism.^2^ What happens to the microvasculature between attacks, when the skin looks normal, has barely been studied. We report one such intercritical observation, obtained with quantitative NVC, in a patient with otherwise typical primary erythromelalgia.

A 40-year-old non-smoking man was referred for microvascular assessment. He had an 8-year history of recurrent burning pain, erythema, and warmth of the hands (Fig. 1A) and feet, triggered by heat and relieved by cooling, fully consistent with primary erythromelalgia. Investigations had ruled out myeloproliferative disease, vasculitis, and connective tissue disorders, and SCN9A sequencing was negative. At the time of NVC, he was clinically asymptomatic, with normal skin colour and temperature at the examined sites, and on stable long-term therapy with low-dose aspirin, gabapentin, duloxetine, and as-needed non-steroidal anti-inflammatory drugs. The last erythromelalgic episode had occurred several months before the examination.

**Figure 1:**
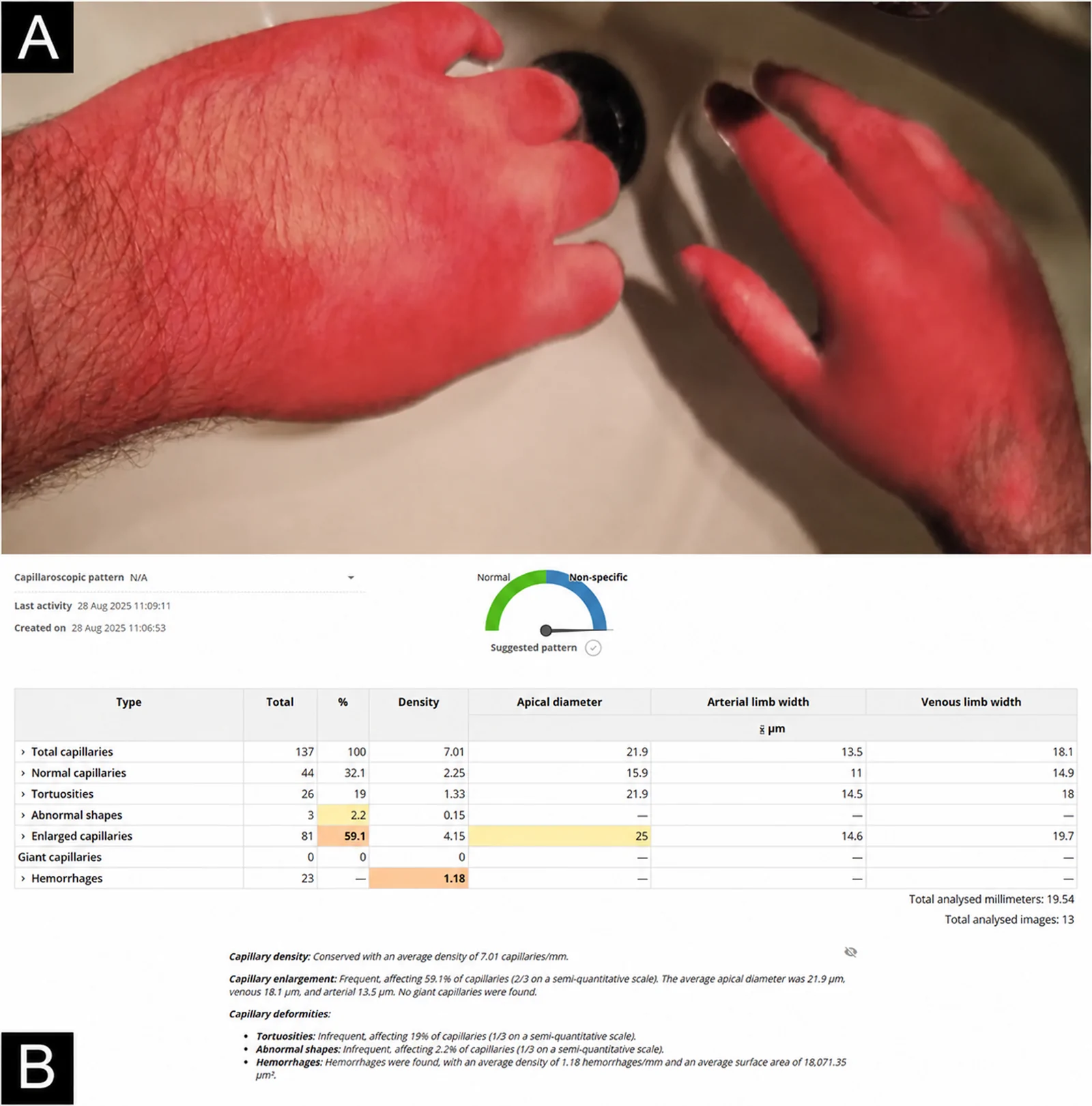
Clinical presentation and quantitative capillaroscopic analysis in a 40-year-old man with primary erythromelalgia. (A) Hands during a typical erythromelalgic episode, showing marked erythema of the fingers; at the time of nailfold videocapillaroscopy the skin was clinically unremarkable. (B) CAPI-Detect quantitative summary of the same patient: 137 capillaries analysed across 13 images, preserved capillary density (7.01 mm), enlarged capillaries in 59.1% of loops, no giant capillaries, and 23 microhaemorrhages; the algorithm classified the pattern as non-specific.

NVC was performed after 15 minutes of acclimatisation at a room temperature of 20 °C to 23 °C, using immersion oil, at 200× magnification with an optical probe videocapillaroscope Horus, according to the standards of the EULAR Study Group on Microcirculation in Rheumatic Diseases.^3^ The second to fifth fingers of both hands were examined, with exclusion of the thumbs, and the periungual skin was free of trauma, recent manicure, manipulated cuticles, or local lesions. Quantitative analysis with the CAPI-Detect machine-learning algorithm^4^ identified dilated or enlarged capillaries without giant capillaries (apical diameter > 20 and < 50 µm) in 59.1% of analysed loops, with 23 microhaemorrhages across 13 analysed images (Fig. 1B). There were no giant capillaries, capillary density was preserved at 7.01 mm, and architecture was orderly, with no neoangiogenesis, ramified capillaries, or avascular areas (Fig. 2A and B). On the EULAR fast-track algorithm, the overall picture is best classified as a non-scleroderma pattern with non-specific abnormalities, given the preserved capillary density and the absence of giant capillaries, avascular areas, and neoangiogenic or ramified capillaries.^5^

**Figure 2:**
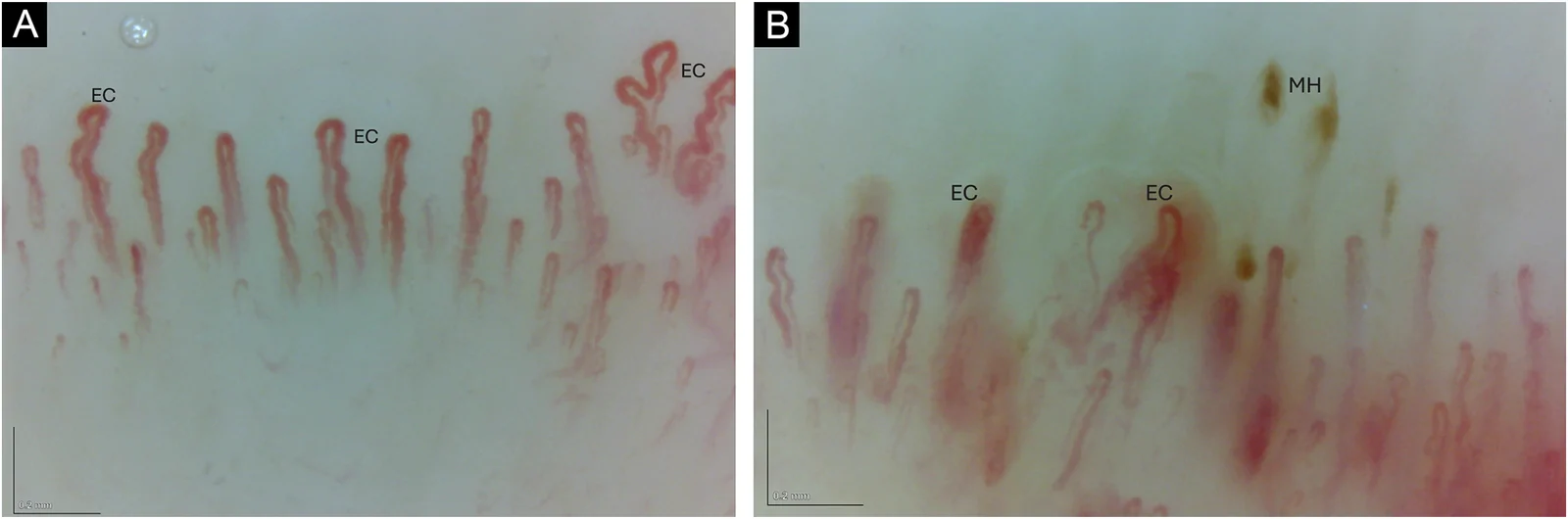
Intercritical nailfold videocapillaroscopy (200× magnification. (A) Multiple Enlarged Capillaries (EC) with preserved density and orderly architecture. (B) Enlarged Capillaries (EC) together with a Microhaemorrhage (MH). Both images correspond to a non-scleroderma pattern with nonspecific abnormalities.

These findings should be interpreted cautiously. The morphology observed, namely dilated or enlarged capillaries without giant capillaries, scattered microhaemorrhages, preserved density, and orderly architecture, is not specific for erythromelalgia. Monticone and colleagues described essentially the same picture, with widened loops, microhaemorrhages, and pericapillary oedema, in patients with acrocyanosis more than two decades ago,^6^ and a recent French multicentre cohort confirmed similar abnormalities in a substantial proportion of acrocyanotic patients.^7^ The experimental work of Mørk and colleagues, by contrast, found reduced capillary density during heat-induced erythromelalgic attacks,^2^ a result in the opposite direction to ours, and earlier case-level reports of intercritical NVC have generally been unremarkable.^1^ The present observation should not be read as a disease-specific signature.

What seems to us worth further investigation is the persistence of quantitatively abnormal capillaroscopy outside the flare. At the time of examination, the patient had no erythema, no warmth, and no pain, yet quantitative NVC detected widened loops and microhaemorrhages. This dissociation differs from acrocyanosis, where capillaroscopic abnormalities accompany a chronically altered skin phenotype. If similar findings can be reproduced in larger series with appropriate comparators, they would raise the possibility that primary erythromelalgia carries a low-grade structural microvascular component, rather than being purely functional. A single case cannot settle this question.

The limitations of this report are clear. The findings come from one patient, and may reflect chance, an unrecognized confounder, or the chronic vasoactive therapy the patient was receiving. Long-term low-dose aspirin and gabapentin can modify cutaneous microcirculation, and duloxetine, a serotonin-noradrenaline reuptake inhibitor, has well-documented vasoactive effects mediated by noradrenergic tone. NVC was performed at a single time point, whereas paired in-crisis and intercritical examinations in the same patient would be needed to separate flare-dependent from persistent components.

What an AI-based quantitative tool adds in this setting is limited to the objective counting of loops, the measurement of diameters, and the enumeration of microhaemorrhages. The algorithm is not designed to classify disease in an individual patient and is not used for that purpose here. The case nevertheless makes a practical argument for a prospective multicentre study of NVC in primary erythromelalgia, with paired in-crisis and intercritical examinations, an acrocyanosis comparator group, ideally drug-naive patients, and a standardized quantitative readout. Only such a design can determine whether persistent dilatation of enlarged capillaries and microhaemorrhages outside attacks represent a reproducible feature of the disease, or simply a non-specific finding shared with other acrosyndromes.

## Research data availability

The entire dataset supporting the results of this study was published in this article.

## Financial support

None declared.

## Authors’ contributions

Angelo Nigro: Conceptualization; data curation; investigation; methodology; writing, original draft; writing, review and editing. The author approved the final version of the manuscript.

## Conflicts of interest

None declared.
